# Comparative genomics of VirR regulons in Clostridium perfringens strains

**DOI:** 10.1186/1471-2180-10-65

**Published:** 2010-02-25

**Authors:** Antonio Frandi, Alessio Mengoni, Matteo Brilli

**Affiliations:** 1 Department of Evolutionary Biology, via Romana 17-19, I-50125 Firenze, Italy; 2 Université de Lyon, F-69000, Lyon, Université Lyon 1, CNRS; INRIA; UMR5558; Laboratoire de Biométrie et Biologie Evolutive, F-69622, Villeurbanne, France

## Abstract

**Background:**

*Clostridium perfringens *is a Gram-positive anaerobic bacterium causing severe diseases such as gas gangrene and pseudomembranosus colitis, that are generally due to the secretion of powerful extracellular toxins. The expression of toxin genes is mainly regulated by VirR, the response regulator of a two-component system. Up to now few targets only are known for this regulator and mainly in one strain (Strain 13). Due to the high genomic and phenotypic variability in toxin production by different strains, the development of effective strategies to counteract *C. perfringens *infections requires methodologies to reconstruct the VirR regulon from genome sequences.

**Results:**

We implemented a two step computational strategy allowing to consider available information concerning VirR binding sites in a few species to scan all genomes of the same species, assuming the VirR targets are at least partially conserved across these strains. Results obtained are in agreement with previous works where experimental validation of the promoters have been performed and showed the presence of a core and an accessory regulon of VirR in *C. perfringens *strains with three target genes also located on plasmids. Moreover, the type E strain JGS1987 has the largest predicted regulon with as many as 10 VirR targets not found in the other genomes.

**Conclusions:**

In this work we exploited available experimental information concerning the targets of the VirR toxin regulator in one *C. perfringens *strain to obtain plausible predictions concerning target genes in genomes and plasmids of nearby strains. Our predictions are available for wet-lab researchers working on less characterized *C. perfringens *strains that can thus design focused experiments reducing the search space of their experiments and increasing the probability of characterizing positive targets with less efforts. Main result was that the VirR regulon is variable in different *C. perfringens *strains with 4 genes controlled in all but one strains and most genes controlled in one or two strains only.

## Background

*Clostridium perfringens *is a Gram-positive anaerobic species able to form heat-resistant endospores and to live in many habitats, from marine sediments to animal gut, to soil. The genus *Clostridium *comprises species causing severe diseases such as botulism, tetanus, gas gangrene and pseudomembranosus colitis that are generally due to the secretion of powerful toxins. *C. perfringens *is the most prolific toxin producer within the genus; several of its extracellular toxins and enzymes have been identified as for instance *α*-toxin (plc, phospholipase C), *β*-toxin (hemolysin family toxin), *ϵ*-toxin, *θ*-toxin (*pfoA*), *κ*-toxin (*colA*, collagenase) and others. Toxins are thought to act synergistically in the development of pathogenesis, and *C. perfringens *strains show a high degree of phenotypic and pathogenic variability, so that understanding the control of the expression of toxin genes is critical to help in fighting diseases caused by this bacterium. The identification of similarities and differences in the set of pathogenic instruments (i.e. genes) of different strains will help to define effective strategies of infection control.

Pathogens usually have precise control mechanisms for toxin production so that expression only takes place when required e.g. when the density of the bacterial population overcomes a certain threshold, or when the bacterium reaches a certain cell-type/organ.

**Figure 1:**
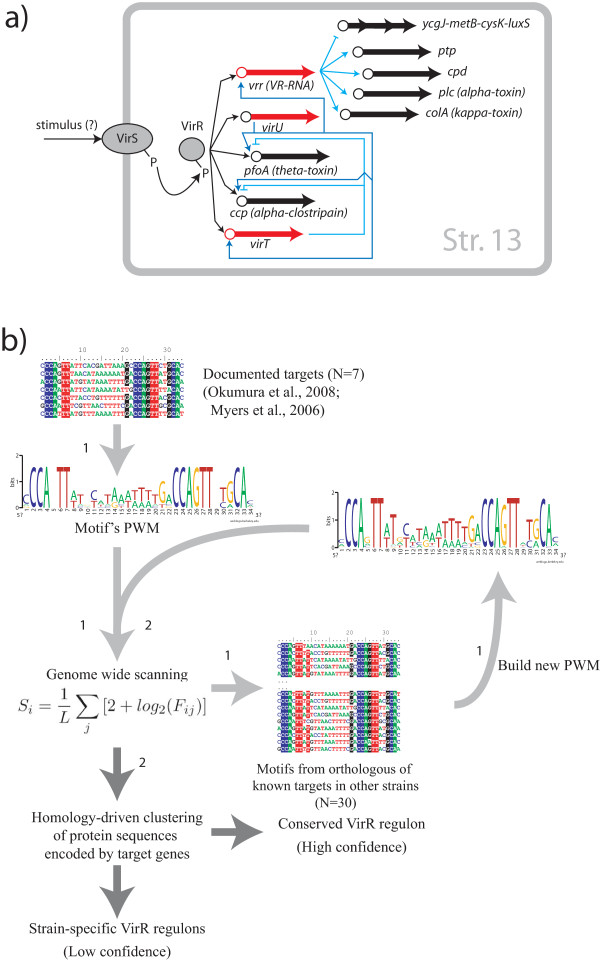
**Biological system and scheme of the strategy**. a) The two component system VirR/VirS and its experimentally validated targets are here schematically represented. Information mainly come from studies performed in Str. 13; modified from [7]. b) Scheme illustrating the two step strategy that allowed to use information coming mainly from a single strain to do the predictions on all other strains in a less biased way. In the first step a position weight matrix (PWM) calculated from a limited number of experimentally validated motifs is used to scan the genomes and to make a list of possible targets. Within that list we looked for sequences corresponding to known targets using clustering, we retrieved their motifs and we obtained a second PWM. This includes the variability of the motif in several strains and was used for the final scan of the genomes.

In bacteria, quorum sensing and environmental signal detection and transduction depend on the activity of dedicated two component systems consisting of a membrane bound sensor histidine kinase and a response regulator. The kinase activity of the sensor is activated by specific signals, triggering phosphorylation of the cognate response regulator. The phosphorylated regulator then actively changes gene expression of its target genes through binding of specific DNA motifs [1]. In *C. perfringens *a major role in integrating environmental signals with virulence competes to the two-component VirR/VirS system, where VirR is the response regulator and VirS the membrane anchored sensor protein [2] (figure 1a). The first VirR regulated promoters have been located upstream of toxin genes [3] and subsequent works showed that VirR target sequences are formed by a pair of imperfect direct repeats, separated by 7-8 nucleotides (depending on how the repeat is defined) [4]. These repeats are known as VirR box1 and VirR box2 (VB1 and VB2) and are located within a core region of about 50 base pairs located immediately upstream of the -35 element of the promoter of regulated genes. The two VirR boxes are both required for VirR mediated transcriptional activation, and mutation of either of them drastically reduces the expression level of target genes. The binding of VirR to its boxes is required for the efficient positioning of the RNA polymerase to the promoter. Furthermore in all the upstream regions of genes directly regulated by VirR, the two boxes are in the same relative position with respect to the promoter and are on the same face of the helix. DNA spacing and helical phasing play a crucial role in the transcriptional activation by VirR, as demonstrated by the insertion or deletion of 5 base pairs in the region between VB1 and VB2 that displaces them on opposite faces of the DNA double helix: in this situation a pronounced reduction of the expression level of genes controlled by VirR was observed [5].

The VirR/VirS regulatory network is not only involved in direct control of toxin encoding genes (figure 1a), but also of several other genes such as *hyp7 *(*vrr*) a gene encoding a regulatory RNA (VR-RNA) which controls the rate of transcription of *colA*, *plc*, *ptp *(protein tyrosine phosphatase) and *cpd *(encoding 2',3'-cyclic nucleotide phosphodiesterase) [6]. A recent paper dealing with the *in silico *identification of VirR regulated promoters in *C. perfringens *str. 13 followed by experimental validation, allowed to identify additional direct VirR targets, namely *virT*, *virU *and *ccp *(*α*-clostripain gene) [7]. The former two genes are particularly interesting because they are regulators of gene expression. Two genes only appeared to be controlled by *virT *(*pfoA *and *ccp*), while *virU *is active with respect to *pfoA*, *ccp*, *hyp7*, and *virT*. A mutational analysis revealed a clear parallel with what observed for *hyp7*, because the gene expression level of their targets is unchanged in *virT *or *virU *nonsense mutants, with respect to the wild-type, allowing to conclude that the functional forms are the *virT *and *virU *RNA [7]. Moreover, three additional genes regulated by VirR and coding for hypothetical proteins, were found in different *C. perfringens *strains: CPF_1074, CPF_0461 in *C. perfringens *ATCC13124 and CPR_0761 in *C. perfringens *SM101 [8]. It is now clear that the two component VirR/VirS system is at the top of a hierarchical regulatory cascade where it directly stimulates the transcription of several virulence-related genes including three different regulatory RNAs that are in turn able to control several other genes [6]. Because of the large heterogeneity in toxin production by *C. perfringens *strains [8], it is interesting to define the genes belonging to the direct VirR regulon in closely related genomes to assess the degree of evolutionary conservation of the VirR regulon. This could also clarify the evolutionary patterns that are at the basis of the divergence between these strains from a common ancestor. However the experimental strategy cannot be easily implemented for all strains, so that it is necessary to integrate information from different strains in a bioinformatics protocol. In this work we extend the bioinformatic approach of [7] to scan the genomes and plasmid sequences of all available genomes of *C. perfringens *strains (Table 1), and identify genes that are putatively controlled by the VirR/VirS system. We implemented a two step strategy allowing to consider information concerning VirR binding sites in all these genomes and defining the core (evolutionary conserved) and accessory (strain-specific) VirR regulons in different strains. Results obtained could help to better define strategies for pathogenicity studies and control strategies in *C. perfringens *and can moreover be used to design focused wet-lab experiments.

**Table 1:** Genomes and plasmids analyzed

C.p. Strain	Type (name)	Sequencing Status	N Genes	Length (nt)
*Str. 13*	G	Finished	2905	3085740
*ATCC 13124*	G	Finished	3066	3256683
*ATCC 3626*	G	Draft	3427	3896305
*C JGS1495*	G	Draft	3254	3661329
*CPE F4969*	G	Draft	3118	3510272
*D JGS1721*	G	Draft	3485	4045016
*E JGS1987*	G	Draft	3729	4127102
*SM101*	G	Finished	2748	2921996
*C. perfringens*	P (pBCNF5603)	Finished	36	36695
*C. perfringens*	P (pCP8533etx)	Finished	63	64753
*F4969*	P (pCPF5603)	Finished	73	75268
*F5603*	P (pCW3)	Finished	51	47263
*F5603*	P (pCPF4969)	Finished	62	70480
*SM101*	P (1)	Finished	10	12397
*SM101*	P (2)	Finished	11	12206
*Str. 13*	P (pCP13)	Finished	63	54310

List of genomes and plasmids used in this study. The *Type *column indicates if a sequence is a genome (G) or a plasmid (P) in that case we also indicate the name of the plasmid within round parentheses. C.p. stands for *Clostridium perfringens*.

## Results and Discussion

### Comparisons of C. perfringens strains

As a preliminary analysis we studied the variability of the selected genomes using both standard phylogenetic techniques and a comparison of all intergenic sequences. The alignment of *rrnA *operons for a total of 4719 nt was used to build a Neighbor-Joining tree revealing that these strains are closely related [Additional file 1: panel a]. In agreement with a low differentiation on ribosomal operon sequences, bootstrap support for the branching pattern was quite low; in fact, 32 variable sites only were found in the alignment, which were evenly distributed between strains [Additional file 1: panel b]. However, the comparison of a large number of intergenic sequences extracted from the genomes revealed that some of them are quite variable between the different strains with respect to the very conserved *rrnA *operon (down to 82% with respect to *C. perfringens *Str. 13, [Additional file 1: panel c]).

### Regulon prediction in sequenced C. perfringens strains

**Figure 2:**
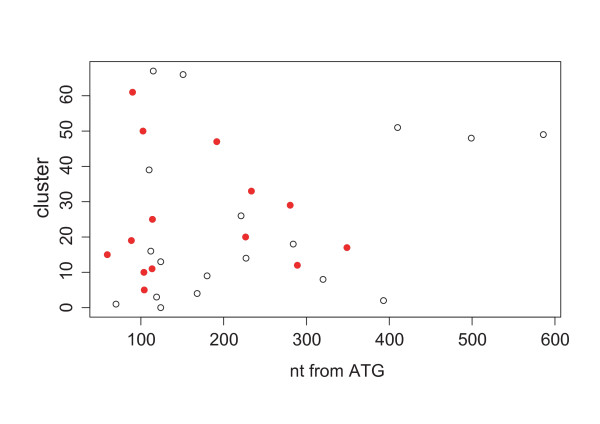
**Distribution of distances from gene**. The distance of the motifs with respect to the translation start site (x-axis) is shown. Motifs are grouped by homology of the downstream gene (cluster identifier is on the y-axis). Most of the targets are located in the first 200 nt from the start of the gene, but some of them (and notably several corresponding to characterized ones) are located at larger distances. Red circles correspond to orthologous groups from Table 2.

The presence of VirR and VirS sequences was checked in all strains using blast and the functionally characterized sequences of Str. 13 as queries. We found that they are indeed both present in all strains and that they are moreover always organized in what resembles a bi-cistronic operon with the two genes often overlapped (data not shown). We scanned available *C. perfringens *genomes using the VirR position weight matrix (PWM) derived from experimental observations, following the procedure reported in figure 1 (see Methods for details). At the time we performed this analysis (April, 2009), the NCBI microbial genome database stored three different complete genomes for *C. perfringens *corresponding to strains 13, ATCC 13124 and SM101, plus the draft genomes of five strains (ATCC 3626, JGS1721, JGS1987, F4969, JGS1495) in the form of whole genome shotguns (Table 1). Despite the higher probability of errors in gene assignments characterizing draft genomes, we decided to include them to expand the scope of our genomic comparison. A whole genome scanning was performed using a PWM derived from the region comprising several experimentally validated VirR binding sites [7,8]. A new PWM was generated from the targets identified in the first scanning by using 30 motifs found in the promoters of genes that are orthologous to known targets and then used for a second genome scanning. In this way we avoid the biases that affect the first matrix, obtained from only a few sequences mainly coming from one strain. After our two-step strategy, we collected all genes with a motif scoring more than 0.88, which is the lowest value observed for an experimentally tested VirR target gene (corresponding to gene CPF_1074, [8]). At this threshold we retained at end 53 occurrences of the VirR motif. Analysis of their location with respect to the start codon of the downstream coding sequence revealed thet most of them are at around 100 bp from the beginning of the gene (figure 2). The larger distance observed for some of the motifs may be due to longer 5' untranslated regions or may account for some different level of regulation for those genes. The list of genes putatively regulated by VirR was splitted in three different groups after clustering similar sequences (see Methods), by defining the: i) conserved VirR regulon as formed by chromosomal genes retrieved in at least two different genomes; ii) the accessory regulon with chromosomal genes present in a single strain; iii) the mobile regulon, including genes found on plasmids.

#### The conserved VirR regulon

The conserved regulon (Table 2), appeared to contain all known target genes [7,8] with the exception of CPR 0761 and *virT*. The former can be identified in the genome of strain SM101 only, while the latter has been found in strain 13 and ATCC3626; in both cases we were able to identify a VirR binding motif in their promoter (Table 3).

**Table 2:** Conserved VirR regulon

Product	Genomes								REF
	ATCC13124	Str.13	SM101	F4969†	JGS1721†	JGS1495†	JGS1987†	ATCC3626†	
***α*-clostripain**	CPF_0840	CPE0846	CPR_0833	AC5_0918	CJD_0991	CPC_0878	AC3_1028	AC1_0991	[7]
*ccp*	1.52	1.52	1.52	1.52	1.52	1.52	1.52	1.52	

**Reg. RNA**	CPF_1204	CPE0957		AC5_1228	CJD_1316	CPC_1202	AC3_1326	AC1_1388	"
*vrr*	1.31	1.31		1.31	1.21	1.31	1.31	1.31	

**Perfringolysin O**	CPF_0156	CPE0163		AC5_0210	CJD_0196	CPC_0186	AC3_0278	AC1_0175	"
*pfoA*	1.18	1.18		1.18	1.18	1.18	1.18	1.18	

**Reg. RNA**	CPF_0925	CPE0920		*	CJD_1073	*	AC3_1102	AC1_1131	"
*virU*	1.20	1.20		1.26	1.26	1.20	1.20	1.20	

**hypothetical**	CPF_1074		CPR_0937	**			**	**	[8]

	0.88		1.11	1.03			1.03	1.03	

**hypothetical**	CPF_0461		CPR_0762					AC1_0537	"

	1.28		1.38					1.28	

**hypothetical**				AC5_0209		CPC_0185			

				1.18		1.18			

**Reg. RNA**		CPE0845						AC1_0990	[7]
*virT*		1.2977						1.29	

Predicted VirR regulons, only genes present in at least two genomes are shown. Numbers below each gene name correspond to the score calculated as described in Methods (on a maximum attainable score of 1.52). As described in the text, most of the known VirR targets belongs to this group. * no open reading frame identified in this region but DNA sequence identical to CPE0920; ** no open reading frame identified in this region but DNA sequence identical to CPF_1074, **†**: draft genomes.

**Table 3:** Strain specific VirR targets

Product	Gene	Score	**Dist**.	Strain
2-keto-3-deoxygluconate kinase	AC3_0259	1.26	124	JGS1987**†**

hypothetical protein AC3_0622	AC3_0622	1.16	70	JGS1987**†**

hypothetical protein AC3_A0724	AC3_A0724	1.04	393	JGS1987**†**

hypothetical protein AC3_A0725	AC3_A0725	1.04	119	JGS1987**†**

conserved hypothetical protein	AC3_A0081	1.11	180	JGS1987**†**

resolvase/recombinase	AC3_0180	1.15	264	JGS1987**†**

put. lipid A export ATP-binding/permease (MsbA)	AC3_0181	1.15	124	JGS1987**†**

hypothetical protein AC3_A0587	AC3_A0587	1.34	227	JGS1987**†**

hypothetical protein AC3_0277	AC3_0277	1.18	112	JGS1987**†**

hypothetical protein AC3_A0194	AC3_A0194	1.25	284	JGS1987**†**

hypothetical protein AC1_A0478	AC1_A0478	0.80	75	ATCC 3626**†**

hypothetical protein AC5_A0236	AC5_A0236	1.04	110	F4969**†**

put. metal-dependent hydrolase	CPR_1028	1.34	499	SM101

hypothetical protein CJD_0545	CJD_0545	0.95	153	JGS1721**†**

hypothetical protein CJD_1387	CJD_1387	1.30	75	JGS1721**†**

Genes identified as VirR targets that are present in a single strain. Strain JGS1987 suggests an expansion of the VirR regulon. **†**: draft genomes.

One target only appeared to be conserved in all tested strains, corresponding to the *α*-clostripain gene. Four genes were shown to be conserved in all strains but SM101. Interestingly, strain SM101 appeared to have the lowest degree of conservation of VirR targets. A search for the corresponding gene sequences in the genome confirmed that they are absent, in agreement with a previous comparative analysis that showed the absence of several virulence factors and toxins and the presence of specific repertoire of genes encoding bacteriocins [8]. On the converse, missing genes in draft genomes cannot be considered as surely absent. Concerning CPE0920 (*virU*) and CPF_1074, corresponding to a regulatory RNA encoding gene and to a gene with unknown function respectively, they have not been identified in some of the genomes, but using their sequences we were able to identify regions with perfect matching using blastn (data not shown) and to locate VirR motifs in their upstream regions (see Table 2). Myers et al. [8] showed that purified VirR is able to bind the promoter of CPR_0761 and of CPF_0461. From our analysis it emerged that CPF_0461 in str. ATCC1324 is the ortholog to CPR_0762 in str. SM101, for which too we predicted the presence of a VirR binding motif upstream. This motif is the same attributed to CPR_0761 and whose ability to bind VirR has been tested by Myers et al., 2006. Our comparative analysis, then suggests that the truly regulated gene could be the latter, because of the conservation of the site upstream of its homologs in two other organisms (ATCC3626 and ATCC1324), while we were not able to find sequences resembling CPR_0761 in any other *C. perfringens *strain by blasting both protein and nucleotide sequences against their genomes. Alternatively, the two genes can also form an operon, with CPR 0761 performing an unknown function.

#### The accessory VirR regulon

We consider this dataset low confidence for two reasons: first of all this group of genes comprises only one experimentally verified target, i.e. *virT *(CPE0845, [7]) and moreover, all other genes have been found in draft genomes only. The list of all putative targets of VirR is shown in Table 3.

Notably, JGS1987 is characterized by an expansion of the VirR predicted regulon, while the accessory regulon of ATCC3626, F4969 and SM101 strains is composed of a single gene. The case of *virT*, a regulatory RNA, is particularly interesting. This sRNA implements a negative feed-back loop on some of the VirR targets i.e. *pfoA *and *ccp *[7]. Our analysis showed that *virT *is present in two strains only (strain 13 and strain ATCC3626). We can thus predict that the other strains lack this negative control and express *pfoA *and *ccp *at different levels eventually by using additional regulations. Actually, strains as ATCC 13124 produces large quantities of gangrene-associated toxins [9] and JGS1987 is a Type E strain which, tough containing an enterotoxin gene (*cpe*), did not show enterotoxin production [10]. The relatively large predicted regulon (10 genes) of JGS1987 may contain genes responsible for its peculiar pathogenicity profile. Within such regulon seven genes code for proteins of unknown function. One of them corresponds to a resolvase/recombinase (AC3_0180) suggesting a possible scenario in which host invasion is linked to gene mobilization. The other two genes with assigned function in the putative regulon of strain JGS1987 include a 2-keto-3-deoxygluconate kinase and a putative lipid A export permease. The first one has been associated with resistance to oxidative stress in *C. perfringens *mutants after transposon mutagenesis [11]. Concerning the putative permease of lipid A, it is known that lipid A is one of the main mediator of bacterial pathogenesis and strongly stimulates in ammation in host tissues [12], so that our prediction is reasonable.

#### The 'mobile' VirR regulon

Our analysis identified three targets located on plasmids, one coding for *ϵ*-toxin (pCP8533etx_p28) in plasmid pCP8533etx from strain NCTC 8533B4D, in addition with two hypothetical proteins, sharing 98% identity, in pCP8533etx (pCP8533etx_p40) and in pCPF5603 (pCPF5603_50) of strain F5603, respectively. Concerning plasmid pCP8533etx, we noticed that it is also present in the shotgun sequences from ATCC3626 (data not shown based on blastn comparisons) and also in that case we were able to find a VirR motif upstream of the gene encoding *ϵ*-toxin.

### Plasmid analysis

**Figure 3:**
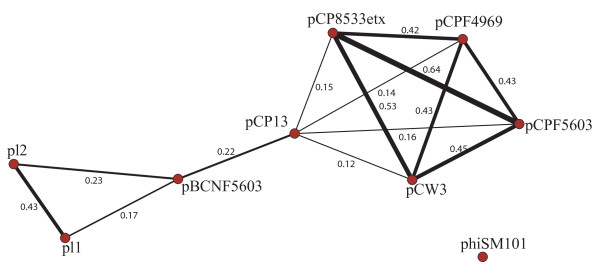
**Plasmid comparison**. Here we applied one of the analysis available in the Blast2Network package [13] consisting in a comparison of plasmid gene content (see Methods for a concise description of the methodology). Values connecting nodes (plasmids) correspond to the percentage of shared genes with respect to the total number of genes of the two plasmids (Jaccard coefficient) and can be considered as a measure of relatedness in terms of evolutionary history (common ancestor) and horizontal transfers and recombination. After this analysis, two groups of plasmids emerged, that are connected through the edge between plasmids pBCNF5603 and pCP13. In the right group of plasmids we identified some VirR targets. The high similar gene content of some of the plasmids in that group may suggest a high rate of horizontal transfer/recombination between different strains, so raising the possibility of the transfer of the VirR targets. Moreover, the connection between the two groups can also suggest that transfers between the two groups of plasmids can happen.

Plasmids can be transferred between species, and gene content similarities between plasmids can be used to trace gene flow between different strains. To evaluate evolutionary relationships relating plasmids from *C. perfringens *species, we performed an analysis to quantify the number of genes shared by each pair of plasmids. For this reason, we built the phylogenetic profiles of the proteomes encoded by plasmids in these strains. The phylogenetic profiles for each group of proteins were obtained by comparing all those proteins one against each other with the package Blast2Network [13]. A phylogenetic profile, or phyletic pattern, is represented by a matrix where each row corresponds to a plasmid molecule and each column to a given protein family. The cell at the intersection between row *i *and column *j *indicates the presence of a component of protein family *j *in plasmid *i*. A phylogenetic profile can be thus interpreted as a graph with two types of nodes: those corresponding to plasmid molecules are connected to nodes of protein families if the corresponding plasmids contains the gene encoding that protein. These matrices can become very large when many plasmids and proteins are involved, so that their analysis and biological interpretation is difficult. A strategy for dimensionality reduction can be through deletion of nodes corresponding to protein families and connection of plasmids directly, through edges that reflect the number of shared protein families (see [Additional file 2] for a scheme). The obtained *hypergraph *is reported in figure 3, where plasmids are connected by links weighted on the basis of the number of common genes. A group of four connected plasmids (i.e. sharing several genes), including pCP8533etx and pCPF5603, was found. This finding is in agreement with previous data showing that plasmids pCPF5603 and pCP8533etx evolved from insertion of mobile genetic elements carrying enterotoxin or *etx *genes, respectively, onto a common progenitor plasmid [14]. This group of plasmids is connected to a second group, composed of three plasmids (plasmid 1, plasmid 2 and pBCNF5603) through a *bridge *represented by pCP13. This implies that pCP13 shares different genes with plasmids from both groups i.e. it may be considered as an evolutionary link between the two groups, one including plasmids with putative VirR targets and the other one with no targets. Plasmids pCP13 and pBCNF5603 seem to have acquired regions from different sources during evolution. Interestingly pCPF5603, belonging to the first group, and pBCNF5603 have been isolated from the same strain, but do not share common regions. The gene encoding the enterotoxin (*cpe*) is only present in pCPF5603 and pCPF4969 and the link from pCP13 to pCP8533etx and pCPF5603 comprises the gene encoding *β*-toxin. These data confirm and extend the detailed analysis performed by [15]. They observed that plasmids pCPF5603 and pCPF4969 share a region of about 35 kb that it is not present in pCP13. From our analysis it emerged that the genes comprised in that region could be conserved also in plasmids pCP8533etx and pCW3.

## Conclusions

In this work we exploited experimental information concerning a small number of promoters controlled by VirR to predict the corresponding regulons in all other *C. perfringens *genomes and plasmids available. Our results are in agreement with previous analysis and suggest that the size of the VirR regulon is quite variable in the analyzed strains as also evidenced by works showing that these strains encode different repertoires of toxin genes. Particularly interesting are the cases concerning *vrr*, *virU *and *virT*, because they encode regulatory RNA that affect gene expression of several other genes. Thus, even at the short phylogenetic distances spanned by these strains [Additional file 1], there could be significant changes in the regulatory cascade initiated by VirR. An event of gain or loss of a VirR target can affect the gene itself only, such as when the event involves a gene coding for a *toxin*, or it can spread downstream of VirR when it involves a regulatory gene, so that also its targets will be affected. As an example consider the regulation exerted by VirR on *virT *in Str. 13 (figure 1a). This gene is present only in Str. 13 and in Str. ATCC3626, where it is regulated by VirR. Experiments have demonstrated that *virT *encodes a small RNA able to repress the expression of *ccp *and *pfoA *and all these genes are positively controlled by VirR. The loss/gain of *virT *or of VirR binding sites in its promoter will thus have an impact on its own expression, but this will propagate downstream to *ccp *and *pfoA*.

The prediction of VirR targets in the genome of strain JGS1987 revealed the presence of 10 specific putative targets that could be important for the peculiar characteristics of this strain.

On an evolutionary perspective, we noticed that once one gene have been found to be regulated by VirR in one genome, it is either regulated by VirR in other genomes or it is lost. This suggests that many of these genes are useful only when controlled by VirR, and also in this case, that their function is not essential for pathogenesis. Then we can imagine that after loss of the VirR binding site these genes are rapidly deleted from the genome; alternatively the deletion may involve both the gene and its promoter. This may happen when the deletion of relatively large genomic regions occurs. Actually, genomes of *C. prefringens *strains have been shown to possess many different genomic islands which may be subjected to frequent events of rearrangemens [8].

## Methods

### Binding sites identification

To identify motifs corresponding to the binding site of VirR we devised the following strategy (illustrated in figure 1b). Using experimentally validated VirR targets (CPE0163, CPE0846, CPE0845, CPE0920, CPE0957 from [7] and CPF_1074 and CPF_0461 from [8]), we derived a position weight matrix describing the region encompassing the VirR box 1 and 2, for a total of 34 nucleotide positions. This matrix was used for a first scanning of whole genome sequences. All the motifs identified upstream of known targets or their orthologs in the other strains were used to build a second PWM that was used for a second round of genome scanning to identify candidate VirR targets. Genome scanning was performed with a sliding window approach from first nucleotide to genome length - L, where L is the motif's length. Each 34-mer was scored using the function proposed in [16]:

where *F*_*ij *_is the frequency of the *i*^*th *^base at the *j*^*th *^position. *S*_*i *_is an information-based measure of potential binding sites. We retained only motifs having a score larger than or equal to the lowest score for an experimentally validated target, corresponding to a threshold of 0.88. Each motif found along the genome was then associated with a gene when located within the region going from 100 nucleotides downstream to 600 nucleotides upstream of the corresponding first codon and on the same strand of the motif.

### Clustering protein sequences

Protein sequences of candidate targets were clustered using the MCL algorithm coupled with Blast2Network [13], whose source code was changed accordingly. Blast2Network performs an all-against-all Blast comparison of protein sequences, followed by translation of the results in an adjacency matrix. From here on we changed the B2N code to allow the use of the MCL with a similarity measure corresponding to the normalized alignment bit score between homologous sequences:

where *S*_*ii *_is the maximal score attainable using the *i*^*th *^query and it corresponds to the query aligned with itself. The adjacency matrix is normalized to make it stochastic, a prerequisite for the MCL algorithm used to define clusters of orthologous sequences. The MCL algorithm simulates flow alternating two algebraic operations on matrices: *expansion *of the input matrix (**M**^**out **^= **M**_in _* **M**_**in**_) models the spreading out of flow and *inflation *(*m*_*ij *_= ). Parameter *r *controls the granularity of the clustering and it is set to 2.

After these two steps we apply diagonal scaling to keep the matrix stochastic and ready for the next iteration. Inflation models the contraction of flow, and it is thicker in regions of higher current and thinner in regions of lower current. The consequence is that the flow spreads out within clusters while evaporating in-between clusters leaving at convergence an idempotent matrix revealing the clusters hidden in the original adjacency matrix.

### Plasmid analysis

Concerning the identification of VirR targets, we analysed plasmids with the same procedure used for genomes. Phylogenetic profiling and the hypergraph describing the similarity in gene contents of different plasmid molecules were calculated using the software Blast2network [13] and visualization with the software Visone [17]. The phylogenetic profiling technique is described in detail in several papers, e.g. [18,19] so that we will not discuss it here in detail, it is enough to say that by comparing the distribution of different genes in different plasmids we can quantify the extent at which proteins tend to co-occur which is an indication of the degree of functional overlapping between different proteins. We want to spend some word concerning the hypergraph shown in figure 3. Let's suppose to have an adjacency matrix describing homologies between proteins encoded by several different plasmids. In this matrix, element *m*_*ij *_corresponds to the similarity between sequences *i *and *j*. However these matrices can be quite large (i.e. the total number of proteins in the study set), so that it is possible to apply some dimensionality reduction approach to extract the information we are interested in. In our case, given the mobility of genes encoded on plasmids, we wanted to assess the degree of similarities between them in term of gene content, and to identify the most plausible routes for gene exchange in the strains under analysis. One way to do that is to calculate the similarity in the phylogenetic profiles of each plasmid and then reduce the original matrix to a new one whose size corresponds to the number of plasmids in the dataset. In this new matrix, the values correspond to the similarity in gene content between every pair of plasmids. Given the binary nature of phylogenetic profiles calculated by B2N, it is possible to to quantify the level of similarity between them using the Jaccard similarity coefficient. Plasmids with highly similar gene content will then give very tight clusters, and plasmids in-between different clusters (sharing some of their genes with plasmids in one clusters and some other genes with an otherwise unrelated cluster of plasmids) could be important because they share genes with different molecules i.e. they could represent preferential routes for the passage of genes between plasmids that are not in contact.

### Alignments and Phylogenetic analysis

The alignment of *rrnA *operons was performed using the software muscle [20] with default parameters. The alignment has a total of 4719 nucleotides, 32 of which are variable, and was used as input to the software mega [21] to build a phylogenetic tree. The algorithm used was the Neighbor-Joining with different rates for transitions and transversions and 100 bootstrap replicates.

### Comparison of intergenic sequences

The comparison of intergenic sequences was performed as follows: all intergenic sequences were extracted from the genome of Str. 13 using gene annotations and were then filtered for a minimum length of 100 nucleotides, obtaining 1633 sequences. These sequences were then blasted against the other genomes. We retained each first blast hit when the e-value of the alignment was less then 1E-06. The boxplots shown in [Additional file 1: panel c] have been obtained for the totality of matches for a genome.

## Authors' contributions

MB wrote ScoreSeq, a Java program to scan full genome sequences with a PWM that is available upon request. MB and AF performed the analysis. AM, MB identified the biological system to be studied, discussed the approach and drafted the paper. All authors participated in manuscript preparation.

## Supplementary Material

Additional file 1 **Comparison between strains**. a) Phylogenetic tree of *rrnA *operons of the eight strains used. Numbers at the nodes indicate bootstrap support on 100 total replicates. The bar at the bottom is in substitutions per site indicating a very low variability of *rrnA *operons. b) Number of differences between strains confirming the previous observation. c) Boxplots summarizing the variability of the intergenic sequences of seven strains with respect to Str. 13. All intergenic sequences were extracted from the genome of Str. 13, filtered to retain only those longer than 100 nt and blasted against the other genomes using an E-value threshold of 1E-06. Click here for file

Additional file 2 **Scheme to obtain the hypergraph shown in Figure **3. Two plasmids encoding 5 and 7 proteins are compared. In the upper panel, the di-graph of plasmids and protein families is shown. This di-graph can be translated in a phylogenetic profile matrix, indicating for each plasmids the protein families they code for. By comparing the two rows corresponding to the two plasmids, by using e.g. the Jaccard coefficient, it is possible to reconstruct the graph of plasmids, connected by links that corresponds to the number of shared proteins with respect to the total number of protein families encoded by these plasmids. Click here for file
