# Supplementary material for: Comparative genomics of VirR regulons in Clostridium perfringens strains
Source: BMC Microbiol. 2010 Feb 25;10:65. doi: 10.1186/1471-2180-10-65 (PMC2838878; doi:10.1186/1471-2180-10-65)

a)

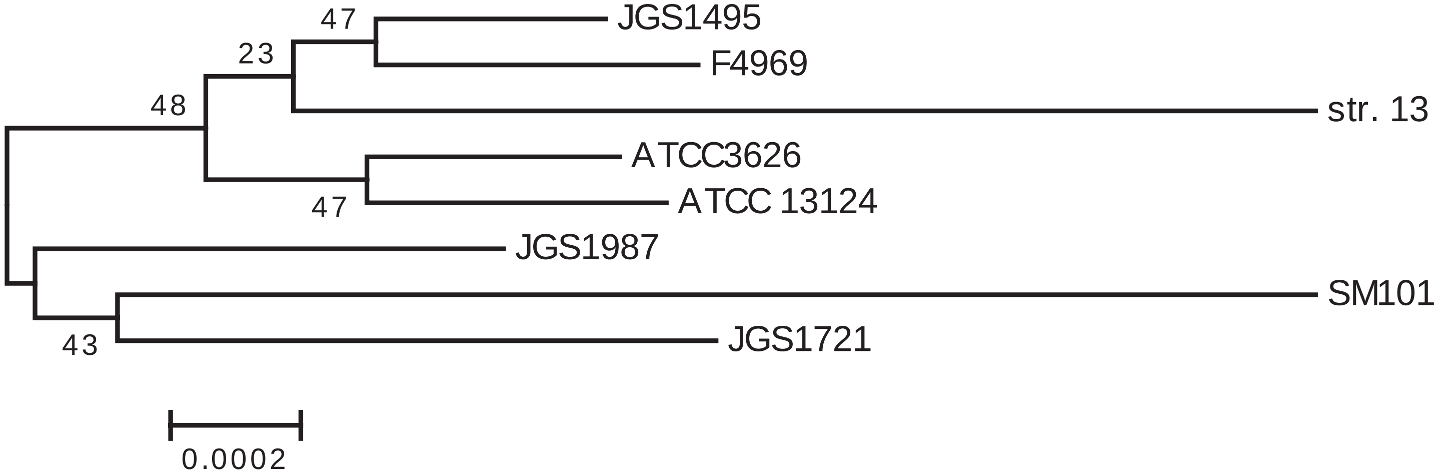

b)

Number of differences

	JGS1721	str._13	JGS1987	ATCC3626	ATCC_13124	JGS1495	F4969
**SM101**	13	20	12	12	14	15	15
**JGS1721**		13	9	9	11	10	10
**str._13**			12	12	12	9	11
**JGS1987**				8	10	7	9
**ATCC3626**					4	7	7
**ATCC_13124**						5	5
**JGS1495**							4

Aligned sequences length ~4800 nt

c)

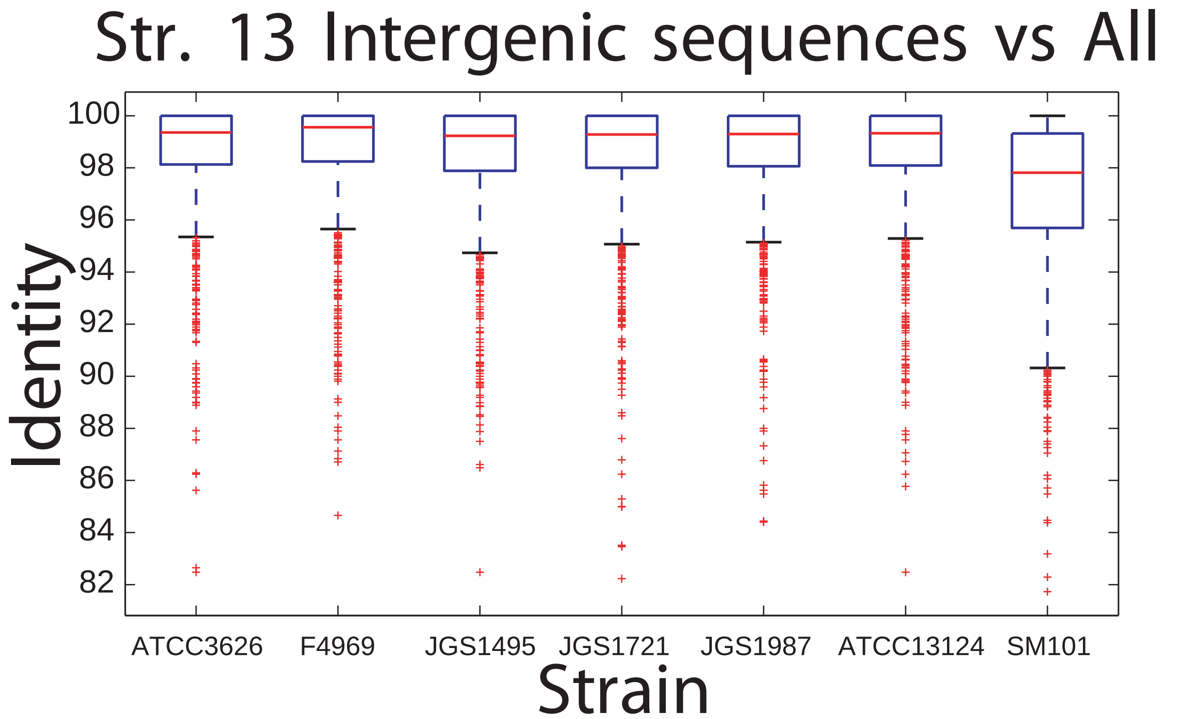

Supplement: Additional file 1 — Comparison between strains. a) Phylogenetic tree of rrnA operons of the eight strains used. Numbers at the nodes indicate bootstrap support on 100 total replicates. The bar at the bottom is in substitutions per site indicating a very low variability of rrnA operons. b) Number of differences between strains confirming the previous observation. c) Boxplots summarizing the variability of the intergenic sequences of seven strains with respect to Str. 13. All intergenic sequences were extracted from the genome of Str. 13, filtered to retain only those longer than 100 nt and blasted against the other genomes using an E-value threshold of 1E-06. [file 1471-2180-10-65-S1.PDF]
