# Supplementary figures and images for: Comparative genomics of VirR regulons in Clostridium perfringens strains
Source: BMC Microbiol. 2010 Feb 25;10:65. doi: 10.1186/1471-2180-10-65 (PMC2838878; doi:10.1186/1471-2180-10-65)

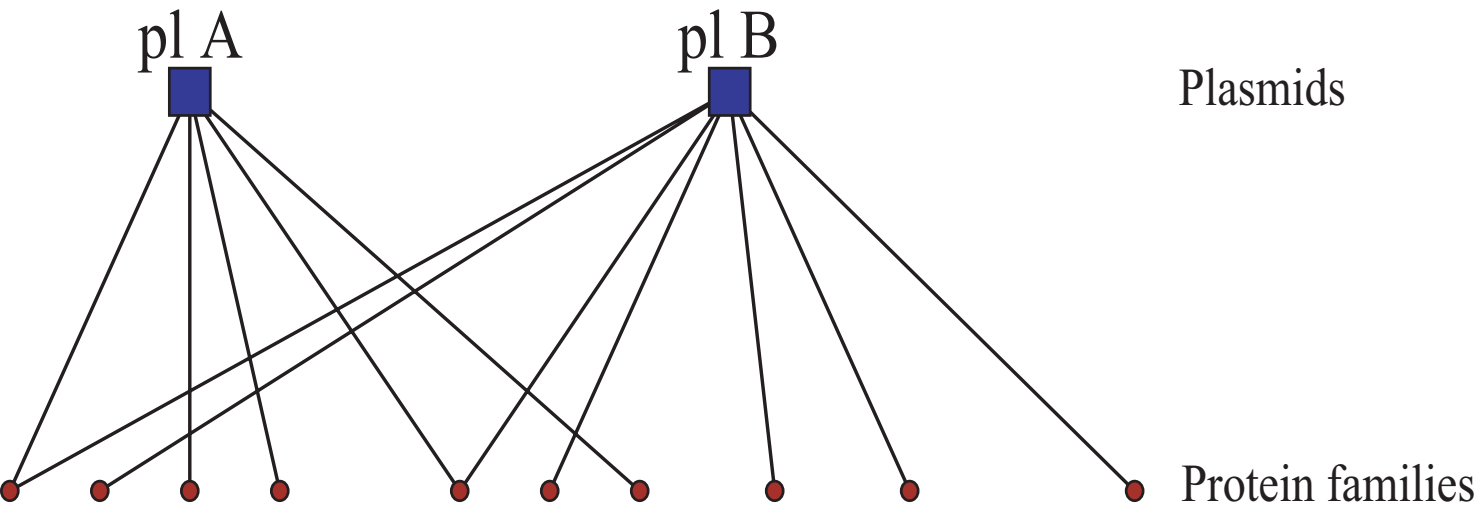

Phylogenetic profiles

|      | p1 | p2 | p3 | p4 | p5 | p6 | p7 | p8 | p9 | p10 |
|------|----|----|----|----|----|----|----|----|----|-----|
| pl A | 1  | 0  | 1  | 1  | 1  | 0  | 1  | 0  | 0  | 0   |
| pl B | 1  | 1  | 0  | 0  | 1  | 1  | 0  | 1  | 1  | 1   |

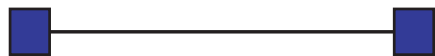

Hypergraph

Supplement: Additional file 2 — Scheme to obtain the hypergraph shown in Figure 3. Two plasmids encoding 5 and 7 proteins are compared. In the upper panel, the di-graph of plasmids and protein families is shown. This di-graph can be translated in a phylogenetic profile matrix, indicating for each plasmids the protein families they code for. By comparing the two rows corresponding to the two plasmids, by using e.g. the Jaccard coefficient, it is possible to reconstruct the graph of plasmids, connected by links that corresponds to the number of shared proteins with respect to the total number of protein families encoded by these plasmids. [file 1471-2180-10-65-S2.PDF]
